# Prevalence and predictors of adult hypertension in Kabul, Afghanistan

**DOI:** 10.1186/1471-2458-14-386

**Published:** 2014-04-23

**Authors:** Khwaja Mir Islam Saeed, Mohammad Hafez Rasooly, Nick JW Brown

**Affiliations:** 1 Surveillance/DEWS Directorate, Afghan National Public Health Institute (ANPHI), Ministry of Public Health, 3rd Floor, Room # 9, Massoud Square, Kabul, Afghanistan; 2 Salisbury District Hospital Visiting Faculty, Salisbury, Wiltshire, UK; 3 Aga Khan University, Karachi, Pakistan; 4 Southampton University Hospitals Trust Visiting fellow, University of Southampton, Southampton, Hampshire, UK

**Keywords:** Prevalence, Associated factors, Blood pressure, Hypertension, Urban, Afghanistan

## Abstract

**Background:**

The prevalence of hypertension is rising worldwide with an estimated one billion people now affected globally and is of near epidemic proportions in many parts of South Asia. Recent turmoil has until recently precluded estimates in Afghanistan so we sought, therefore, to establish both prevalence predictors in our population.

**Methods:**

We conducted a cross-sectional study of adults ≥40 years of age in Kabul from December 2011-March 2012 using a multistage sampling method. Additional data on socioeconomic and lifestyle factors were collected as well as an estimate of glycaemic control. Bivariate and multivariable analyses were undertaken to explore the association between hypertension and potential predictors.

**Results:**

A total of 1183 adults (men 396, women 787) of ≥ 40years of age were assessed. The prevalence of hypertension was 46.2% (95% CI 43.5 – 49.3). Independent predictors of hypertension were found to be: age ≥50 (OR = 3.86, 95% CI: 2.86 – 5.21); illiteracy (OR = 1.90, 1.05 – 1.90); the consumption of rice >3 times per week (OR = 1.43, 1.07 – 1.91); family history of diabetes (OR = 2.20, 1.30 – 3.75); central obesity (OR = 1.67, 1.23 – 2.27); BMI ≥ 30 Kg/meter squared (OR = 2.08, 1.50 – 2.89). The consumption of chicken and fruit more than three times per week were protective with ORs respectively of 0.73 (0.55-0.97) and 0.64 (0.47 – 0.86).

**Conclusions:**

Hypertension is a major public health problem in Afghan adults. We have identified a number of predictors which have potential for guiding interventions.

## Background

High blood pressure is at epidemic levels globally. An estimated 1 billion people are affected [1-3] with particularly high prevalence in parts of South Asia thought to be a result of a combination of genetic susceptibility and lifestyle transition [4,5]. Predictors of hypertension in High Income Countries (HIC) include family history, age, race, obesity, physical inactivity, lack of exercise, cigarette smoking, excessive salt intake and excessive alcohol intake [2,3].

In Afghanistan, due to years of conflict, it has been impossible to make any robust estimates of non-communicable disease prevalence. There is, however, no reason to believe that Afghans are less susceptible to non-communicable disease and estimates are therefore urgently required. The purpose of this study was to estimate the prevalence of hypertension and assess the predictors in urban Afghan adults.

## Methods

We conducted a cross-sectional study between December 2011 and March 2012 to estimate the prevalence of hypertension and its associated factors in Kabul. All adults aged 40 years and over who had been residing for at least one year or more in the 17 districts of Kabul city were eligible. The city has approximately 3,289,000 inhabitants living in 17 residential districts made up of neighborhoods (*Gozar*) comprised of 534,900 households [6]. We requested the Kabul municipality to provide a list of all neighborhoods and their representatives and were able to study 13 districts. To balance considerations of cost, resources, and time without compromising the representativeness of the sample, a two-phase cluster sampling technique was used as follows. Eligible subjects were selected by multistage sampling: in the first stage, a sample of neighborhoods was selected randomly from each district. In the second stage, a main *masjid* (mosque) was selected as a hallmark and heads of households around the *masjid* were asked to approach the team settled there. Finally, one adult from each household was randomly selected and interviewed after written consent was obtained.

Sample size estimation was based on a prior specified precision level of 5% and the assumption that the proportion of potential risk was similar to other studies conducted in other similar settings. We adjusted for intracluster correlation and concluded that 1,200 participants would be required.

One member from each household aged 40 years and over was interviewed using a proforma designed for the study. Height and weight were measured by measurement tape and electronic weighing scale. Using standard international criteria, we defined a BMI of ≥ 30 kg/m2 as obese and a BMI of 25–30 kg/m2 as overweight. A BMI of 18.5 to 25 kg/m2 was considered normal. A waist circumference of ≥ 94 cm for men and ≥ 80 cm for women were considered centrally obese [7,8].

Blood pressure was measured by twice by android sphygmomanometer in the sitting position on the left arm. Before analysis, blood pressure was dichotomized to hyper- or normotension by either systolic of ≥ 140 mmHg, or diastolic of ≥ 90 mmHg, or both. Those already on treatment were considered hypertensive irrespective of our own readings. Fasting blood sugar was tested using portable glucometer once in the morning before breakfast and analysed by the glucose oxidase method (check-ref). Data was single entered and, after cleaning, analysis was undertaken on 1,183 individuals [9]. Data on potential covariates such as: age, sex, ethnicity, family history of the disease, educational status, income, residential area, obesity, diabetes mellitus, smoking status, snuff using, physical activity and dietary behavior were collected through the structured pretested and modified questionnaire. No questions were made about alcohol as Afghanistan is an Islamic state and alcohol is considered a narcotic and is illegal. For normally distributed variables, we calculated mean and Standard Deviation (SD). For categorical variables we used chi-Squared and logistic regression and derived both uni- and multivariate estimates of Odds Ratios (OR) and 95% Confidence Interval (95% CI). Data were analyzed using SPSS 20 [10].

Ethical approval was obtained from the Institutional Review Board (IRB) of the Ministry of Public Health, Afghanistan.

## Results

### Descriptive data (Table 1)

**Table 1:** Frequency distribution of the demographic, socioeconomic characteristics and behavior factors of study participants (n = 1183)

**Variables**	**Numbers (%)**
**Age in years**	
40 – 49	691 (59.3)
50 – 59	242 (20.7)
60 – 69	159 (13.6)
70 and over	74 (6.3)
**Sex**	
Males	396 (33.5)
Females	787 (66.5)
**Level of education**	
Illiterate	657 (57.1)
Primary/Unofficial education	118 (9.9)
Secondary school	216 (18.3)
High school and more	174 (14.7)
**Monthly income**	
≤ 10000	524 (52.4)
10000 – 20000	293 (29.3)
20000 – 30000	80 (7.3)
≥ 30000	103 (10.3)
**Work status**	
Government employee	260 (22.7)
Business	59 (5.2)
Jobless	63 (5.5)
Unable to work	472 (41.3)
Housewife	290 (25.3)
**Knowledge of blood pressure**	
Yes	195 (16.5)
No	988 (83.5)
**Smoking status**	
Current smoker	61 (5.2)
Ever smoker	95 (8.1)
Never smoker	1018 (86.7)
**Basic mass index**	
Underweight	13 (1.1)
Normal weight	346 (29.6)
Obese	365 (31.2)
Overweight	445 (38.1)
**Type of cooking fat**	
Solid cooking fat	821 (69.6)
Liquid cooking fat	339 (28.6)
Other	4 (1.8)
**Frequency of eating meat in a month**	
< 3 times per month	307 (26.3)
3 – 6 time per month	592 (50.4)
6 – 9 times per month	160 (13.6)
≥ 9 times per month	116 (9.9)
**Frequency of eating rice in a month**	
< 3 times per month	536 (49)
3 – 6 time per month	432 (39.5)
6 – 9 times per month	126 (11.5)
**Frequency of taking vegetable in a month**	
Once a week	240 (20.3)
Twice a week	291 (24.6)
Thrice a week	166 (14)
>3times a week	486 (41.1)
**Frequency of taking fruits in a month**	
Once a week	181 (15.3)
Twice a week	257 (22.6)
Thrice a week	233 (19.7)
>3times a week	502 (42.4)
**Mode of transportation to job**	
Walk by foot	747 (63.2)
Bicycle	25 (2.1)
Motorcycle	15 (1.3)
Car	72 (6.1)
Public transport	59 (5)
**Frequency of physical activity**	
< 10minutes per day	1022 (86.4)
10 – 30minutes per day	132 (11.2)
≥ 30minutes per day	29 (2.4)
**Frequency of sitting in hours**	
< 10hours per week	238 (20.1)
10 – 30hours per week	812 (68.8)
≥ 30hours per week	131 (11.1)
**Frequency of walking in hours**	
< 10hours per week	375 (31.9)
10 – 30hours per week	625 (53.1)
≥ 30hours per week	176 (15)
**Blood pressure**	
Normotensive	573 (48.5)
Pre-Hypertensive	218 (18.4)
Hypertensive	392 (33.1)
**General obesity**	
Yes	379 (32)
No	804 (68)
**Central obesity**	
Yes	687 (58.8)
No	481 (41.2)

**Figure 1:**
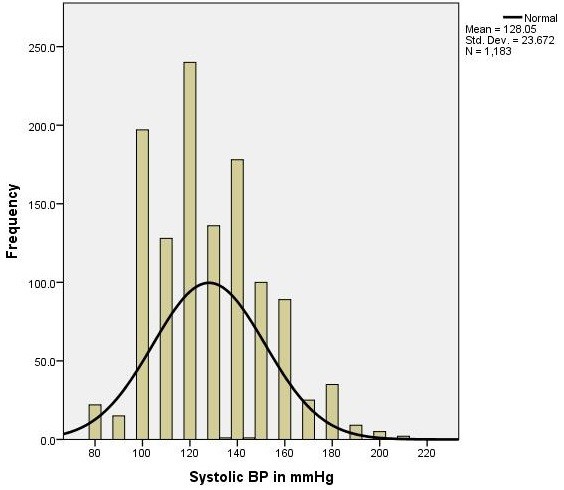
Graphic (histogram) distribution of systolic blood pressure of study participants (1183) aged ≥ 40 years in Kabul city, 2012.

**Figure 2:**
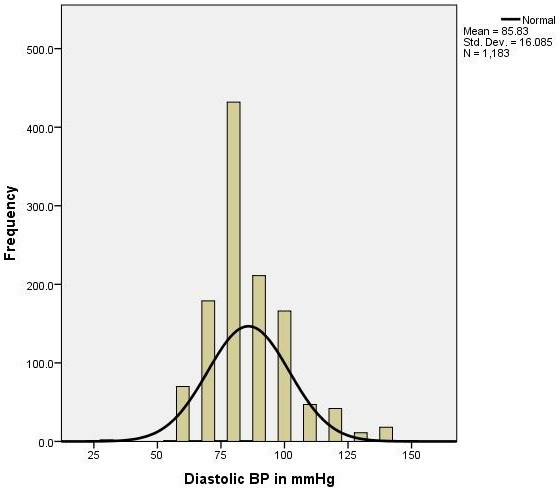
Graphic (histogram) distribution of diastolic blood pressure of study participants (1183) aged ≥ 40 years in Kabul city, 2012.

The mean age of the study subjects was 49.5 years and most (59.3%) were aged between 40 and 50 years. Sixty eight percent of the participants were ethnic Tajik and 20.7% were ethnic Pashtuns. Two thirds were female. Most study participants were illiterate with a monthly income of 2,000 to 6,000 Afghanis (AFN) (One USD = 50AFN). Forty percent of participants were unemployed or were casual laborers, while 25% of respondents were women engaged in home employ. Twenty two percent were government employees and the rest were engaged in the agricultural industry as farmers. Approximately 3.3% of respondents were retired and too old to engage in physical labor. 83% of participants had no knowledge of hypertension but only 14% were smokers. The prevalence of either systolic or diastolic hypertension was 46.2% (95% CI 43.5 – 49.3). The distribution of both systolic and diastolic blood pressure can be reviewed in Figures 1 and 2.

### Multivariate analysis

Hypertension was associated with age but not gender. Literacy was protective (OR for illiteracy = 1.90 95% CI: 1.05 – 1.90 and P value <0.05) but level of income, smoking status and knowledge of diseases were not predictive. Unemployment and inability to work predicted hypertension ORs = 2.04 (95% CI: 1.49 – 2.78) and 1.69 (95% CI: 1.20 – 2.38) respectively. The results for bivariate analysis can be reviewed in Table 2 and for multivariate analysis in Table 3.

**Table 2:** Bivariate analysis of bio-demographic, socio economic and behavior risk factors associated with hypertension among Kabul citizens (n = 1183)

**Variables**	**Hypertensive**	**Non-hypertensive**	**Odds ratio**	**% 95 CI**
**Age in years**				
40 – 49	231 (33.4)	460 (66.6)	1	Reference
50 – 59	143 (59.1)	99 (40.9)	2.87	2.13 – 3.88
60 – 69	112 (70.4)	47 (29.6)	4.74	3.26 – 6.90
70 and over	55 (74.3)	19 (25.7)	5.76	3.43 – 9.94
**Sex**				
Males	178 (44.9)	218 (55.1)	1	Reference
Females	368 (46.8)	419 (53.2)	1.07	0.84 – 1.37
**Level of education**				
literate	363 (53.8)	312 (46.2)	2.06	1.63 – 2.61
Illiterate	183 (36.0)	325 (64.0)	1	Reference
**Monthly income**				
≤ 200USD	247 (47.1)	277 (52.9)	1.07	0.85 – 1.35
>200USD	299 (45.4)	360 (54.6)	1	Reference
**Work status**				
Government employee	90 (34.6)	170 (65.4)	1	Ref
Business	21 (35.6)	38 (64.4)	1.04	0.57 – 1.88
Famer	28 (44.4)	35 (55.6)	1.51	0.86 – 2.64
Jobless	245 (51.9)	227 (48.1)	2.04	1.49 – 2.78
Housewife	137 (47.2)	153 (52.8)	3.37	1.67 – 6.81
Unable to work	25 (64.1)	14 (35.9)	1.69	1.20 – 2.38
**Knowledge of blood pressure**				
Yes	94 (48.2)	101 (51.8)	1	Ref
No	452 (45.7)	536 (54.3)	0.9	0.66 – 1.23
**Smoking status**				
Current smoker	23 (37.7)	38 (62.3)	0.72	0.42 – 1.22
Ever smoker	51 (53.7)	44 (46.3)	1.37	0.90 – 2.10
Never smoker	465 (45.7)	553(54.3)	1	Reference
**Snuff using**				
Current user	58 (55.2)	47 (44.8)	1.51	1.01 – 2.26
Ever user	13 (52.0)	12 (48.0)	1.32	0.59 – 2.93
Never user	469 (45.0)	574 (55.0)	1	Reference
**Walking by foot to work station**				
No	214 (49.3)	220(50.7)	1.22	0.96 – 1.55
Yes	331 (44.3)	416 (55.7)	1	Reference
**Going by car to work**				
No	523 (47.1)	587 (52.9)	1.89	1.14 – 3.15
Yes	23 (31.9)	49 (68.1)	1	Reference
**Going by public Transport to work**				
No	528 (47.0)	596 (53.0)	2.01	1.14 – 3.55
Yes	18 (30.5)	41 (69.5)	1	Reference
**Diabetes mellitus**				
No	455 (44.3)	571 (55.7)	1	Reference
Yes	91 (58.0)	66 (42.0)	1.7	1.23 – 2.43
**General obesity cut off BMI = 30**				
No	324 (40.3)	480 (59.7)	1	Reference
Yes	222 (58.6)	157 (41.4)	1.73	1.23 – 2.43
**Central obesity B**				
No	169 (35.1)	312 (64.9)	1	Reference
Yes	370 (53.9)	317 (46.1)	2.15	1.69 – 2.73
**Frequency of eating rice in a month**				
≤ 6 times per month	439 (45.4)	529 (54.6)	1	Reference
>6 times/month	44 (34.9)	82 (65.1)	1.54	1.05 – 2.27
**Frequency of walking per week in hours**				
< 10 hours per week	202 (53.9)	173 (46.1)	1	Reference
10-30 hours/week	274 (43.8)	351 (56.2)	0.66	0.51 – 0.86
≥ 30 hours/week	68 (38.6)	108 (61.4)	0.50	0.37 – 0.77

**Table 3:** Multivariable analysis risk factors associated with hypertension among Kabul citizens (n = 1183)

**Variables**	**Adjusted OR**	**% 95 CI**	**P Value**
**Age group**			
≤50	1	Reference	-
>50	3.86	2.86 – 5.21	<0.001
**Education level**			
Literate	1	References	-
Illiterate	1.90	1.05 – 1.90	<0.05
**Having rice as meal**			
≤3times a week	1	References	-
>3times a week	1.43	1.07 – 1.91	<0.05
**Having chicken as meal**			
≤3times a month	1	References	-
>3times a month	0.73	0.55 – 0.97	<0.05
**Family history of diabetes**			
No	1	References	-
Yes	2.20	1.30 – 3.75	<0.05
**Taking fruits with meal**			
≤twice a week	1	References	-
>twice a week	0.64	0.47 – 0.86	<0.05
**Central obesity**			
No	1	References	-
Yes	1.67	1.23 – 2.27	<0.001
**General obesity as BMI cut off 30**			
No	1	References	-
Yes	2.08	1.50 – 2.89	<0.001

## Discussion

Our study shows a prevalence of hypertension in Kabuli adults of close to 50%, which is comparable to that in many HIC settings. Predictors included illiteracy, obesity, low levels of physical activity and a family history of diabetes. A higher dietary quality (measured by higher chicken and fruit intake) had a modest protective effect but given the attenuation in the multivariate model it is possible that this association is simply a marker for other social factors. In keeping with the natural history of hypertension, prevalence increased with age.

Strengths of the study include the breadth of sampling across the city and two stage methods.

Our study is unique in Afghanistan in which any research over the last 15 years has been near impossible as a result of chronic warfare and instability.

Limitations of the study include the sampling. For logistical reasons our study was household rather than community based and, therefore, less likely to be involved in regular work. It is possible that the participants were less active or different in other ways to their employed counterparts and may, therefore, not have been representative of all Kabuli adults. We would have liked to undertake more comprehensive assessment of glucose tolerance but were precluded by budgetary constraints.

Our findings were consistent with work in similar settings. In rural Nepal, Khan showed that a high BMI and low socio-economic status were associated with increase odds of hypertension [11]. Other studies in Iran, Turkey, Sri Lanka, Angola and China found similar findings that older age, high blood glucose level, lower level of education, retirement/unemployment and higher body mass index were significantly associated with hypertension [12-17].

Literacy was an independent predictor of hypertension but the association between education and health is a complex one. Though in some settings it may be a marker of (and confounded by) socio economic status, there is no doubt that literacy enhances health awareness an association that has been demonstrated in numerous studies. To this effect, literacy was selected as a target for the Millennium Development Goals by the WHO and UN [11,18,19]. In Afghanistan, as a result of political forces the provision of universal literacy has been complex and challenging. This has been most marked for girls but, at the time of writing, the country is for the first time holding independent elections and we can only hope that education is marked as a priority for the new government.

The other predictors, sedentary lifestyle, obesity and impaired glucose tolerance are all potentially amenable to intervention at a public health level. Sedentary lifestyles may stem from a lack of activity in school. If education were to be available universally and physical activity a mandatory part of the school day, there is every reason to believe that this can be inculcated from an early age for life. Though there may be cultural sensitivities, they can be overcome as Almas and colleagues demonstrated in an innovative study in urban Pakistan [20]. Other than education, better use of the media to inform the population has a role particularly as most households now have at least a television. Subsidising blood pressure checks in primary medical care would additionally encourage update of screening. We feel that, given our findings, both mass and targeted intervention would be feasible. We have shown that Afghanistan is equally at risk of hypertension and has the same risk factors as other populations. As the conflict subsides, our attention must turn to another threat, that of the non-communicable diseases epidemic.

## Conclusion

Though instability has hitherto precluded detailed study, this unique study has shown that hypertension and obesity is at epidemic proportions in household adults in Kabul. As Afghanistan enters a new era, intervention is now a Public Health priority.

## Competing interests

There is no financial or non-financial competing interest of authors in this paper.

## Authors’ contributions

KMIS has been involved in conception, design, and implementation and reporting of the study. MHR has been involved in analysis and report writing while NB has reviewed the whole paper including analysis and interpretations. He has copy edited the manuscript to get ready for publication. All authors read and approved the final manuscript.

## Pre-publication history

The pre-publication history for this paper can be accessed here:

http://www.biomedcentral.com/1471-2458/14/386/prepub
